# Improving institutional platforms for evidence-informed decision-making: getting beyond technical solutions

**DOI:** 10.1186/s12961-022-00948-6

**Published:** 2023-01-16

**Authors:** Jeffrey Mecaskey, Ben Verboom, Marco Liverani, Rhona Mijumbi-Deve, Nasreen S. Jessani

**Affiliations:** 1 HPI Institute, Berlin, Germany; 2 University of Oxford, Oxford, United Kingdom; 3 Department of Global Health and Development, London School of Hygiene and Tropical Medicine, London, United Kingdom; 4 School of Tropical Medicine and Global Health, Nagasaki University, Nagasaki, Japan; 5 The Center for Rapid Evidence Synthesis (ACRES), Kampala, Uganda; 6 Africa Centre for Evidence, University of Johannesburg, Johannesburg, South Africa; 7 Centre for Evidence Based Health Care, Stellenbosch University, Cape Town, South Africa; 8 Department of International Health, Johns Hopkins University, Baltimore, MD United States of America

**Keywords:** Evidence-informed decision-making, Institutionalization, Political economy, Broker, Intermediary, Citizen panel, Knowledge translation, Evidence use, Accountability, Network

## Abstract

Purely technical interventions aimed at enhancing evidence-informed decision-making (EIDM) have rarely translated into organizational institutionalization or systems change. A panel of four presentations at the Health Systems Global 2020 conference provides a basis for inference about contextual factors that influence the establishment and sustainability of institutional platforms to support EIDM. These cases include local structures such as citizen panels in Uganda, regional knowledge translation structures such as the West African Health Organization, global multilateral initiatives such as the “One Health” Quadrapartite and regional public health networks in South-East Asia. They point to the importance of political economy as well as technical capability determinants of evidence uptake and utilization at institutional, organizational and individual levels. The cases also lend support to evidence that third-party (broker and intermediary) supportive institutions can facilitate EIDM processes. The involvement of third-party supranational organizations, however, poses challenges in terms of legitimacy and accountability.

## Introduction

The imperative to advance evidence-informed decision-making (EIDM) has never been greater [1]. In an era marked by competing claims to “truth” and gross misinformation, calls for rigorous evidence to inform the public discourse and global health action have intensified [2]. And yet a growing body of literature asserts that promoting evidence uptake in policy and practice entails much more than merely technical solutions [3–5]. Indeed, it is increasingly recognized that health policy processes (including the generation and use of evidence to inform policy) are shaped by institutional and other contextual factors, including formal structures [6, 7] and relationships [8–10] as well as tacit norms and political culture [5]. One important influencing factor is the system of governance in which policy options are discussed and decisions are made—whether, for example, it is a national, subnational, regional or global system. Crucial to the policy process is also the power of the different actors involved, including the power of ordinary citizens and their ability to engage with evidence-informed policy dialogue.

In this commentary, we aim to contribute to the debate on the role of institutions, organizational arrangements, and broader contexts in supporting EIDM in global health. Drawing on four cases derived from a panel discussion on this topic presented at the Sixth Global Symposium on Health Systems Research, we describe good practices and key challenges in EIDM as a basis for considering how institutional development can contribute to and inform more responsive global health policy and practice. The cases represent different contexts, and yet they all serve to illustrate the complexity of data sharing and evidence uptake, the importance of capabilities associated with the generation and use of evidence, and the role of other contextual determinants including political economy and the wider health sector infrastructure. We summarize the contextual characteristics, scope and key insights for each of the case studies in Table 1 to illustrate the breadth of a set of highly divergent circumstances that inform this debate. The motivation for this commentary reflects our appreciation of the need for detailed, context-specific intelligence as the foundation for effective EIDM.

**Table 1:** Major elements of the case studies

	Regional public health networks in South-East Asia	Citizen panels in Uganda	Regional capacity-building in West Africa	Multilateral collaboration for One Health in Africa
Focus	Cross-border collaboration in infectious disease control	Voice and accountability in public policy	Maternal, newborn & child health policy	Intersectoral coordination for animal, environmental and human health
Locus	Mekong Basin	Uganda	ECOWAS region	WHO African Region
Scope	Regional public health cooperation	Reform of teachers’ professional code of conduct	Use of evidence in MNCH policy-making	Intersectoral surveillance, preparedness and response
Stakeholders	National governments, health sector managers and workers, communities, international organizations	Education Service Commission and educationists	ECOWAS Member State ministries of health, health researchers, international partners	WHO/African Region Member States’ ministries
Brokers	ADB, ASEAN, Rockefeller Foundation, WHO SEARO, WHO WPRO	ACRES	WAHO	United Kingdom FCDO
Primary intervention	Support to cross-border cooperation for infectious disease prevention and control	Empowering grassroots stakeholders to enable participation in high-level policy-making processes	Coordination and coproduction of tailored knowledge transfer strategies in each Member State (e.g. training, norm shifting, networking, top-down changes to organizational procedures)	Sensitization of stakeholders and training on priority competencies related to One Health
Major insights	International brokering institutions can facilitate the sharing of public health data and information across borders to address common health issues	Citizen panels are a pathway that creates trust enough for citizens’ direct input into a high-level policy process	Effectiveness of individual and organizational capacity-building for EIPM is limited in the absence of enabling institutional and political environments	Institutional as well as capacity and interpersonal considerations inform development of priorities in each country
Major challenges	Interoperability, regulatory barriers, sustainability & capacity constraints,	Sustaining these pathways as a more regular approach to public policy engagement	Technical capacity, political leadership and durability of organizational evidence “cultures”	Technical & governance capacity, divergent sectoral priorities

*ACRES* Center for Rapid Evidence Synthesis, *ADB* Asian Development Bank, *ASEAN* Association of Southeast Asian Nations, *ECOWAS* Economic Community of West African States, *EIPM* evidenced-based policy-making, *FCDO* Foreign, Commonwealth & Development Office, *MNCH* maternal, newborn and child health, *WAHO* West African Health Organization, *WHO SEARO* WHO Regional Office for South-East Asia, *WHO WPRO* WHO Regional Office for the Western Pacific

In the four sections that follow, we introduce and describe each of our cases—regional public health networks to facilitate data sharing in Southeast Asia, citizen panels to engage grassroots citizens in policy-making in Uganda, regional integration and capacity-building to support evidence-informed policy-making in West Africa, and multilateral collaboration for One Health in Africa. We close this commentary by weaving together the key insights, lessons and challenges that emerged from the four cases, and providing recommendations for improving EIDM in global health.

## Regional public health networks in Southeast Asia—promoting data and information sharing across borders

The Mekong subregion in Southeast Asia has historically been a hotspot for the emergence and transmission of infectious diseases. Over the past two decades, substantial progress has been made to strengthen disease prevention and control capacities. However, important regional challenges remain, including those associated with artemisinin-resistant malaria, avian influenza, tuberculosis and other endemic diseases that are less prominent on the global health agenda such as cholera and rabies [11]. In recognition of these common challenges, several regional networks have been established to promote the sharing of public health data and information for routine disease surveillance and during outbreaks. For example, the Mekong Basin Disease Surveillance (MBDS) is an innovative network that was established in 2001 by the health ministers of Cambodia, China, Lao People's Democratic Republic, Myanmar, Thailand and Viet Nam to build a regional infrastructure for infectious disease control through mutual learning and support [12]. The Asian Development Bank (ADB) has also sponsored several projects to promote regional health cooperation, such as the Greater Mekong Subregion Communicable Disease Control (GMS-CDC) network in Cambodia, Lao People's Democratic Republic and Viet Nam (www.gms-cdc.org). In addition, vertical programmes have been developed to strengthen regional response to specific diseases, including HIV [13], avian influenza [14] and malaria [15]. While these initiatives have been characterized by differing approaches and configurations, they have all involved activities to promote data sharing at regional meetings or through regular communication between health authorities in bordering countries.

Over time, these efforts have contributed to an unprecedented increase in the circulation of health data, information and expertise in the region, at times leading to joint outbreak investigations. However, a recent study of these networks also identified challenges to data sharing due to gaps between national information systems, different rules to govern the information flow inside and outside the countries and imbalances in technical capacities [16]. As the study reported, concerns with the reliability of the information source may affect international cooperation for routine surveillance and stifle the potential for collective action in the event of emergencies. Further, the same study highlighted the important role of a third-party organization in data-sharing processes since this can provide a central unit to facilitate the transfer, management and dissemination of shared data throughout the network. The involvement of a third-party organization also has the potential to provide an institutional platform to develop regional standards and a legal framework to regulate ethical issues that may arise from data sharing.

In Southeast Asia, recent experiences in regional health cooperation illustrate significant involvement of third-party brokers such as the WHO regional offices, the ADB and the Association of Southeast Asian Nations (ASEAN). However, the institutional landscape is fragmented, with little coordination between overlapping initiatives funded by different donors [17]. Today, ASEAN is in a good position to play a leading role in regional health affairs and act as a catalyst for the diverse range of regional health programmes, including data-sharing activities. The ongoing progress towards the establishment of the ASEAN Economic Community is an important upgrade in regional cooperation which will likely prompt increasing regulatory convergence on several trade issues, with potential spillover effects in public health policy. Yet the achievement of effective collaboration between ASEAN Member States in public health, as in other policy areas, will require a higher level of institutionalization at the national and supranational level and reform of financing mechanisms to address chronic budget issues [18].

## Capacity-building in West Africa—institutional championing and high-level leadership

This case describes the knowledge transfer work of the West African Health Organization (WAHO), which is a specialized health agency of the Economic Community of West African States (ECOWAS). During the past decade WAHO has become increasingly engaged in the promotion of evidence use for health policy-making at the national and regional levels, focusing particularly on strengthening the research use capacity of West Africa’s 15 national ministries of health [19–22]. The organization’s most recent strategic plan prioritizes “improv[ing] the production, dissemination and utilization of health information and research within the ECOWAS region”, including developing “mechanisms for regular dissemination and utilization of knowledge, evidence and information” [22]. Under the umbrella of the donor-funded Moving Maternal, Newborn and Child Health Evidence into Policy (MEP) Project, WAHO’s research unit developed and implemented a knowledge transfer platform (KTP), a series of interventions designed to strengthen the links between evidence and national health policy in West Africa.

Through the platform, WAHO’s research unit and the organization’s Member States work together to “coproduce” and implement tailored, context-sensitive intervention packages to address the specific knowledge transfer needs of individual countries. Interventions vary from in-depth capacity-building trainings on research communication and critical appraisal, and the establishment of networking fora to facilitate engagement and relationship-building between decision-makers, academics and other stakeholders, to the development of formal guidelines to support ministries of health in the complex process of accessing and (appropriately) applying evidence to decisions.

However, while the importance of capacity strengthening, relationship-building and technical and procedural guidance should not be underestimated, a realist case study examining the development of WAHO’s KTP pointed to another factor of at least equal importance: institutions. WAHO’s research unit recognized that previous efforts to strengthen the use of evidence have often had disappointing results, and they argued that this was, at least in part, because of a failure to address issues at what is sometimes called the “institutional level” [23, 24]. Study participants asserted that technical skills and tools to support evidence uptake, while necessary, are not sufficient to achieve ideals of “evidence-informed” policy-making without high-level endorsement from health system leaders, and that in the absence of the institutionalization of “norms of evidence”, lasting improvements in research use are unlikely to be achieved.

In this spirit—and following much behind-the-scenes work by evidence champions within WAHO’s research unit—in 2017, the ECOWAS Assembly of Health Ministers unanimously approved a Resolution on the Use of Evidence, which acknowledged that “a significant amount of research is conducted and that very few findings are used in policy and practice” and called for the use of research evidence by Member States in “the development of health care policies, plans, standards and protocols” [25]. Of course, the long-term effects of such endorsements of EIDM at the highest levels of health governance are as yet unknown. What appears clear, however, is that meaningful change will take time and persistence, and that technical solutions are almost certainly not enough.

## Citizen panels in Uganda—involving frontline workers in policy-making

Questions abound about appropriate approaches to engaging citizens and including their voices in policy-making, particularly in high-level policy processes. Citizens, unlike other stakeholders, may generally be less informed about a particular policy issue, or their voices may be unheard, especially with the use of approaches like citizen representatives, yet input from their lived experience is crucial to inform policy development and successful policy implementation and evaluation. In view of this, the Center for Rapid Evidence Synthesis (ACRES) at Makerere University, Uganda, piloted an experimental project involving citizen panels as a platform for empowering citizen to participate in high-level policy-making processes.

ACRES was built with the aim of supporting policy- and decision-making with high-quality, relevant and timely evidence. It used citizen panels to provide an evidence-informed response to Uganda’s Education Service Commission in its consideration of policy reform options related to the teachers’ professional code of conduct [26]. Ordinary citizens—in this case educationists, including teachers and education managers—of different educational levels, ages and backgrounds were invited to the panel. The selection of eligible participants was conducted through a three-step sampling process (stratified-random-purposive), which involved the mapping of different categories that should be represented (including underrepresented categories such as teachers working in remote rural areas) and random sampling within each category. The participants took part in two citizen panels, a month apart [27, 28]. The two panels lasted half a day each, and served different purposes within the longer decision-making process but involved the same citizens and were moderated by an experienced public systems expert.

Because citizens may lack detailed information about the policy problem, the first panel allowed them to acquire and process knowledge that would permit meaningful contribution to the debate. This knowledge was applied in the second panel in which they engaged with the problem and contributed their views about potential policy solutions.

Most participants were keen to be involved and contribute their views and experiences. There was a response rate of 89% (16 out of 18 invitees) for the first panel and 87.5% (14 of the 16 participants from the first panel) for the second panel. Citizens’ engagement increased in the second panel, as they reported feeling more at ease and more valued simply by being invited to the process a second time. Organizing a second panel, therefore, was crucial to enhance trust and build confidence in the participants. What emerged clearly was the importance of giving citizens adequate background information and time to understand the policy options and their wider implications. However, this experience also showed that spontaneous and unbiased feedback from participants must be encouraged throughout the process, with minimal external “control” and direction (including control from the panel moderators).

In sum, this case highlights the value of citizen panels as an effective method to enable democratic participation in decision-making processes and therefore increase their legitimacy. Following this experience, further citizen panels were convened to inform other policy processes, including community management of the follow-up phase of multidrug-resistant tuberculosis (MDR-TB) in Uganda. Based on findings from a study designed to describe the adherence patterns of MDR-TB patients undergoing directly observed therapy (DOT) supervision at two health facility categories during an intensive phase of treatment [29], citizen panels were convened to develop an understanding of the key factors determining the adherence patterns observed in the descriptive study. Participants included former and current TB patients and their carers, community members and providers of care.

Despite the great promise of these exercises, questions remain about their sustainability and institutionalization. Thus, ACRES and partners are involved in assessing this methodology further to determine contextual factors that affect its effectiveness, uptake and acceptance in different settings and contexts.

## Multilateral collaboration for One Health—working across sectors and interests

Events ranging from the 2014–2016 Ebola virus disease outbreak in West Africa to the ongoing global novel coronavirus (COVID-19) pandemic underscore the importance of focusing on the intersection of animal, human and ecosystem health, or One Health. The One Health quadripartite, a collaboration between the Food and Agriculture Organization (FAO), Organization for Animal Health (OIE), the United Nations Environment Programme (UNEP) and WHO, provides Member States normative guidance for evidence-informed policy and programming with emphasis on sub-Saharan Africa since 2019, with the inclusion of UNEP in 2022 [30]. Despite some success in antimicrobial resistance surveillance, this initiative has yet to see tangible impact on evidence-informed policy and practice [31].

As part of the United Kingdom government’s support for strengthening pandemic preparedness and response, Tackling Deadly Diseases in Africa (TDDA) is a United Kingdom Foreign, Commonwealth & Development Office (FCDO flagship programme to increase the ability of partner countries to prevent and respond to health emergencies. Led by DAI Global, TDDA works closely with the Africa Centres for Disease Control and Prevention and the agencies of the One Health Quadrapartite to provide additional support for adaptation and uptake of evidence-based norms related to strengthening public health services, surveillance, cross-border coordination, communication and behaviours that affect the spread of disease in African countries that are particularly vulnerable to disease outbreaks.

In its examination of the reasons for slow uptake of evidence-informed policy guidelines, TDDA applied comparative policy analysis (CPA) and political economic analysis (PEA) in Cameroon, Chad, Côte d’Ivoire, Mali, Niger and Uganda to assess alignment of sectoral policies in each country as well as patterns across the six countries. A problem-driven PEA identified the major actors, incentives and interests in each country that shape space for change. These analyses informed mapping of the intra- and inter-sectoral challenges as a basis for identifying opportunities for bridging observed performance gaps.

Findings from these analyses revealed a need for capacity-strengthening within and across sectors. Underinvestment in institutions and systems led to gaps in the structures supporting evidence-based policy and programme alignment [32]. Divergent sectoral mandates inhibited effective coordination. Differing and, at times, conflicting interests and incentives of stakeholders served as barriers to change. These common themes notwithstanding, the country-specific circumstances of institutional, organizational and systems opportunities and challenges underscored the imperative of fully contextualized approaches to evidence adaptation and uptake [32].

These analyses also identified opportunities for subtle realignment of inter-sectoral engagement that could contribute significant impact. Shifts in country structures for coordination, for example, rotation among participating ministries, helped ensure more response programming and greater political buy-in [33]. Fuller communication of ministerial priorities and greater understanding of respective interests are likely to increase functional cooperation and performance [34]. These approaches contributed to strengthening EIPM; still, the challenges to optimizing One Health in integrated health security and realizing its promise in practice remains a work in progress.

## Discussion

The case examples discussed in this paper are drawn from diverse geographical and cultural contexts—from West and Central Africa to Uganda to South-East Asia—and a variety of decision-making venues—from regional bureaucracies and national governments to local communities. Yet, some common threads run through these stories, providing lessons that can be used to inform the development of well-designed institutional platforms for linking knowledge and action for health.

First, the cases highlight and contribute to the understanding that supply-side and demand-side factors have to be addressed in equal measure in evidence-informed health policy and practice. Early efforts to link evidence with policy tended to emphasize the supply side, with researchers being encouraged to gain skills to push their findings into decision-making [35]. However, an unequipped demand side is unable to request evidence appropriately, use the evidence they are provided with well or take advantage of available opportunities at the nexus of research and policy. These cases highlight the necessity for the demand side to be empowered—with structure, knowledge and skills—in order for evidence to make a meaningful contribution to the decision-making process and agenda.

Second, the cases also highlight the importance of institutional brokering structures that serve as intermediaries. These intermediaries are crucial in filling in knowledge, skill and resource gaps, for example, evidence synthesis skills and time, that the demand and supply sides may be missing to enable optimal engagement and exchange. This in turn highlights the need for both individual and institutional capacity-strengthening to provide sustained and sustainable structures and systems for data and other evidence sharing and synthesis, analysis and utilization.

Third, the cases also provide a better understanding of the role of knowledge brokerage, especially as performed by institutions (as opposed to individuals). While the traditional roles of knowledge generators (like academics and other researchers), and intended users (like policy-makers), have been well defined, the role of knowledge brokers, entrepreneurs and other intermediaries has been less clearly explored and understood [36], though this has begun to change recently [37–42]. Cases presented here provide lessons for knowledge brokerage in practice and provide clear roles for these institutions, contributing to a growing body of evidence in support of these. However, they also illustrate that brokerage can take place at different institutional levels and between a multiplicity of actors, and that the ideal set-up is likely to be highly dependent on the context and circumstances. Whereas in the case of WAHO and disease surveillance networks in South-East Asia, knowledge brokerage between countries is critical, the case of the One Health quadripartite platform shows that brokerage between sectors is sometimes equally important. The role of institutional knowledge brokers in building and forging relationships between knowledge stakeholders to allow for fruitful interaction and harnessing of their defined contributions to the process is clearly shown here.

While the case studies reflect a set of substantially different settings, a number of additional common themes are apparent. First, context is everything. While global norms and guidelines to facilitate the sharing and uptake of evidence in health policy and practice are increasingly available, recommendations and best practices are often decontextualized or depoliticized, with insufficient consideration of the underlying system of institutions, structures and norms which can direct and shape EIDM processes. This may be influenced by approaches to education and training that encourage simplistic and overly broad notions of research generalizability. However, as we have seen, a better understanding of context is not a secondary consideration, but is central to determining whether, how and what kind of evidence is useful for informing policy-making, and the institutional processes and structures most likely to facilitate its uptake.

Second, political economy matters. The politics of evidence is well established [43, 44]. However, in technically driven areas—like the maternal, newborn and child health continuum of care, One Health and even disease surveillance—the role of institutional and organizational alignment, stakeholder interests and incentives are often less considered [45]. By developing a deeper understanding of the characteristics and impact of these contextual determinants, stronger structures to support evidence uptake can be designed and sustained [46].

Third, the imperative of achieving better health for all must stay at the centre of institutional knowledge transfer efforts. The targeted outcomes of these institutional measures—from increased evidence use by decision-makers, to improvements in data-sharing practices and greater citizen engagement in policy processes—are not just ends in themselves, but possible *means to the ends* of improving the functioning, fairness and responsiveness of health systems and, ultimately, achieving the goal of longer and healthier lives. Evidence use in policy-making can take many forms and serve a number of functions, including instrumental problem-solving, learning and enlightenment, and providing ammunition for political debate and policy deliberation [47]. Many—if not most—of these forms of evidence use do not represent the kinds of problem-driven EIDM that might be expected to lead to downstream improvements in health services and outcomes. When designing and evaluating institutional structures and procedures for EIDM, crude notions of evidence “uptake” should be avoided. We must not lose sight of the higher-order ideal of using research to improve health.

The methods applied here also allow us to challenge some of the known and applauded approaches in EIDM or improve them. For example, the use of citizen panels challenges the use of citizen representatives—a widely used approach—where research has even found that it leaves those represented less informed and not much more empowered, may distort their input along the knowledge transfer continuum, and is affected by their relationship with leadership [48].

Lastly, a focus on the importance of institutions should not obscure the fact that institutions are complex adaptive systems, and therefore unintended consequences are inevitable. Purposive intervention into the workings of complex social systems always produces unanticipated effects [49]; institutional action to support EIDM is no different. Institutional reforms have short-, medium- and long-term ripple effects, and it is imperative that we remain particularly attentive to the possible adverse effects of efforts to systematically integrate evidence into decision-making. For example, institutional procedures and mandates might well work to increase the flow of data and the uptake of research by health sector officials, but they might also risk promoting selective and unsystematic uses of evidence by officials whose incentive structures are impacted by such reforms. Scholars of public sector management have long understood such performative responses to institutional requirements [50]. More broadly, suppose strict protocols for EIDM are institutionalized as standard routine practice in the health sector, from the central structures of the Ministry of Health to provincial and district health departments. How might this constrain the creativity and capacity for innovative problem-solving of decision-makers? And how can the most positive potential outcomes of EIDM be preserved while incorporating local, tacit knowledge in a meaningful way? All institutional change has potential downsides; clear-eyed acknowledgment of this fact can facilitate the recognition and mitigation of unintended harm.

## Conclusion

These case examples spoke to a variety of institutional platforms—both organizational and regional—to enhance EIDM, and yet some important common lessons emerged, pointing to three significant tendencies in the uptake of evidence-informed policy. Although embedded in different contexts, these cases illustrate a continuum of key principles highlighting the complexity of data sharing and evidence use, the crucial role of capabilities associated with evidence generation and use, and other circumstantial determinants, including political economy, the wider health sector infrastructure, and the institutional context. Together, these observations highlight the importance of assessing other contextual domains of evidence—history, social and political change, and institutional alignment—in efforts to advance evidence-informed policy. They also point to the importance of brokers to marshal these allied domains and the limitations of a purely technical approach to EIDM. Most importantly, these stories reveal that capacity-building interventions and other technical solutions, while important, cannot be expected to generate sustainable improvements in EIDM in the absence of meaningful institutional change.

## Data Availability

The datasets used and/or analysed in the case studies are available from the authors on reasonable request.

## Abbreviations

ACRES: Center for Rapid Evidence Synthesis
ADB: The Asian Development Bank
ASEAN: Association of Southeast Asian Nations
CPA: Comparative policy analysis
ECOWAS: Economic Community of West African States
EIDM: Evidence-informed decision-making
FAO: Food and Agriculture Organization
FCDO: United Kingdom Foreign, Commonwealth and Development Office
GMS-CDC: Greater Mekong Subregion Communicable Disease Control
KTP: Knowledge transfer platform
MBDS: Mekong Basin Disease Surveillance
MEP: Moving Maternal, Newborn and Child Health Evidence into Policy Project
MDR-TB: Multidrug-resistant tuberculosis
OIE: Organization for Animal Health
PEA: Political economic analysis
TDDA: Tackling Deadly Diseases in Africa
UNEP: United Nations Environment Programme
WAHO: West African Health Organization
